# Safety issues of tirzepatide (pancreatitis and gallbladder or biliary disease) in type 2 diabetes and obesity: a systematic review and meta-analysis

**DOI:** 10.3389/fendo.2023.1214334

**Published:** 2023-10-16

**Authors:** Qingyue Zeng, Jiao Xu, Xingyu Mu, Yi Shi, Hong Fan, Shuangqing Li

**Affiliations:** ^1^ General Practice Ward/International Medical Center Ward, General Practice Medical Center, West China Hospital, Sichuan University, Chengdu, Sichuan, China; ^2^ Department of Pulmonary and Critical Care Medicine, West China Hospital, Sichuan University, Chengdu, China

**Keywords:** dual agonists, incretin based therapy, tirzepatide, type 2 diabetes, obesity, glucagon-like peptide-1 receptor agonists

## Abstract

**Purpose:**

A systematic review and meta-analysis was conducted to synthesize the available data from clinical trials and assess the safety issues of tirzepatide (pancreatitis and gallbladder or biliary disease) in type 2 diabetes (T2D) and obesity.

**Methods:**

A systematic search was conducted in three electronic databases, namely Embase, PubMed, and the Cochrane Library, up until March 1, 2023, to identify randomized controlled trials (RCTs) comparing tirzepatide to either placebo or active hypoglycemic drugs in individuals with T2D and obesity. Heterogeneity was assessed using the I2 value and Cochran’s Q test, and a fixed effects model was employed to estimate the safety profile of tirzepatide. The safety outcomes of interest, including pancreatitis, the composite of gallbladder or biliary diseases, cholecystitis, and cholelithiasis and biliary diseases, were evaluated. (The composite of gallbladder or biliary diseases incorporated cholelithiasis, cholecystitis, other gallbladder disorders, and biliary diseases.)

**Results:**

A total of nine trials with 9871 participants (6828 in the tirzepatide group and 3043 in the control group) that met the pre-specified criteria were included. When compared to all control groups consisting of basal insulin (glargine or degludec), selective GLP1-RA (dulaglutide or semaglutide once weekly), and placebo, an increased risk of pancreatitis was not found to be significantly associated with tirzepatide (RR 1.46, [95% CI] 0.59 to 3.61; I2 = 0.0%, p = 0.436). For gallbladder or biliary disease, the composite of gallbladder or biliary disease was significantly associated with tirzepatide compared with placebo or basal insulin (RR 1.97, [95% CI] 1.14 to 3.42; I2 = 0.0%, p = 0.558), but not with the risk of cholelithiasis, cholecystitis or biliary diseases.

**Conclusion:**

Based on the currently available data, tirzepatide appears to be safe regarding the risk of pancreatitis. However, the increased risk of the composite outcome of gallbladder or biliary diseases observed in RCTs warrants further attention from physicians in clinical practice.

**Systematic review registration:**

https://www.crd.york.ac.uk/PROSPERO, identifier CRD42023412400.

## 1 Introduction

The escalating prevalence of obesity and type 2 diabetes (T2D) has resulted in significant disability, exorbitant complications and even reduced life expectancy (1). Since 1975, the prevalence of obesity has almost tripled, with a parallel increase in the incidence of T2D (2).

Obesity is the strongest risk factor for T2D as it causes insulin resistance, a key driver in development of T2D (3). Numerous metabolic complications, including but not limited to increased cardiovascular risk, hypertension, dyslipidemia, obstructive sleep apnea, and non-alcoholic fatty liver disease (NAFLD), are commonly observed in individuals with both obesity and T2D (3, 4).

Given the strong association between obesity and T2D, weight loss in people with T2D may have favourable effects on glycaemic control, insulin sensitivity and cardiovascular-metabolic comorbidities (3, 5). However, the fight against obesity has proven to be an arduous endeavour with significant obstacles. The current approach to the treatment of obesity revolves around the implementation of a carefully balanced diet combined with consistent physical activity. However, it is important to recognise that only a small proportion of people with T2D are able to achieve and maintain sustained weight loss (6).

In recent years, glucagon-like peptide-1 receptor analogues (GLP-1 RAs) has revolutionised the treatment of T2D, as they have shown remarkable efficacy in reducing blood glucose levels while promoting substantial weight loss and weight maintenance, allowing for improved disease management (7, 8). GLP-1 RAs also improve multiple cardiometabolic risk factors, reduce risk of cardiovascular events and cardiovascular mortality and have renoprotective effects in people with T2DM (9). However, the efficacy of GLP-1 RAs may be limited, particularly in reducing body weight in people with T2DM, and the occurrence of adverse events such as nausea and vomiting is often influenced by the dosage administered (10, 11). As a result, a considerable number of people with T2DM are not able to achieve the metabolic, and weight loss targets with the currently existing therapies (12).

Glucose-dependent insulinotropic polypeptide (GIP) is the other major incretin hormone (13). Glucagon-like peptide-1 (GLP-1) and glucose-dependent insulinotropic peptide (GIP) are recognized as primary incretin hormones (14). GLP-1 is produced and released by enteroendocrine L-cells located in the distal ileum and colon, whereas GIP is mainly secreted by enteroendocrine K-cells located in the proximal small intestine and is responsible for most of the insulinotropic incretin effect (15, 16). The synergistic activation of GIP and GLP-1 receptors has been recognized as a promising therapeutic approach in the treatment of T2D and obesity (17, 18).

Tirzepatide, a novel dual agonist of GIP and GLP-1 receptors, is currently undergoing development for the management of T2D and obesity (19). Tirzepatide is a synthetic linear peptide molecule that consists of 39 amino acids. Its structure is derived from GLP-1, GIP, and semaglutide, and through the incorporation of two non-encoded amino acid residues, located at positions 2 and 13, and the addition of a C20 fatty acid molecule at position 20, Tirzepatide has exhibited a prolonged half-life and a high affinity towards albumin (20–22).Tirzepatide received its first approval in the US in May 2022 for the improvement of glycaemic control in adults diagnosed with T2D, when used in combination with dietary changes and physical activity. The usual therapeutic doses of tirzepatide are 5mg, 10mg and 15mg. It has greater affinity for GIP receptors than for GLP-1 receptors, while its t½ of approximately 5 days allows once-weekly subcutaneous administration (21). Compared with GLP-1RAs, tirzepatide enhances glycemic control and weight loss to a greater extent (23) can improve both markers of beta cell function and insulin sensitivity together with reduced glucagon secretion (24). Furthermore, it has also demonstrated significant improvements in adipose tissue, lipoprotein metabolism, blood pressure, and cardioprotective effects (25–28).

To date, only one systematic review and meta-analysis has examined the association between tirzepatide and pancreatitis and cholelithiasis, and its results were limited to cholelithiasis and did not include other biliary or gallbladder diseases such as cholecystitis and cholangitis. In addition, recent researches on tirzepatide were not included in this analysis (29–31). The objective of this meta-analysis is to assess the safety concerns of tirzepatide, specifically regarding the potential risk of pancreatitis and gallbladder or biliary disease, in individuals with T2D and obesity. This analysis will gather all relevant evidence from randomized controlled trials (RCTs) to provide a comprehensive assessment of these safety issues.

## 2 Methods

### 2.1 Protocol

Our systematic review and meta-analysis was executed and reported in accordance with the Preferred Reporting Items for Systematic Reviews and Meta-Analyses (PRISMA) statement (32). The protocol was registered in PROSPERO (CRD42023412400).

### 2.2 Search strategy

We conducted a systematic search of three electronic databases - namely, Embase, PubMed, and the Cochrane Library - to identify relevant literature published in English up to March 1, 2023. No limitations were imposed on geographical location or publication type. Our search strategy employed a combination of keywords and medical subject headings (MeSH) to ensure comprehensive coverage of the literature. Specifically, we used the following search terms: (diabetes OR diabetes type 2 OR diabetes mellitus type 2 OR non-insulin dependent diabetes OR T2D OR T2DM OR type 2 diabetes) OR (obesity, obese or overweight) AND (tirzepatide) OR LY3298176) AND (randomized controlled trial). The search strategy was adjusted to meet the requirements of each database.

### 2.3 Inclusion and exclusion criteria

Our review focused on RCTs of tirzepatide that lasted at least 24 weeks and compared its safety with that of other hypoglycemic agents or placebo in patients with T2D and obesity. We excluded review articles, letters, conference abstracts, case reports, non-human studies, editorials, commentaries, expert opinions, non-RCTs and meta-analyses. Two independent reviewers screened citations and assessed full-text publications for eligibility. This approach ensured that only high-quality studies were included in our meta-analysis.

### 2.4 Outcome measures of safety

Our study evaluated the safety of tirzepatide with regard to two major adverse events: pancreatic adverse events and the composite of gallbladder or biliary diseases, cholecystitis, cholelithiasis, and biliary diseases. (The composite of gallbladder or biliary diseases incorporated cholelithiasis, cholecystitis, other gallbladder disorder, and biliary diseases.)The study outlines the process for determining the presence of gallbladder or biliary disease as follows: any occurrences of biliary colic, cholecystitis, or other potential events associated with gallbladder disease should be assessed and further diagnostic tests conducted, if necessary. To diagnose acute pancreatitis, at least two of the following three criteria must be met: the presence of abdominal pain, serum amylase (total and/or pancreatic) and/or lipase levels at least three times the upper limit of normal (ULN), and characteristic findings of acute pancreatitis on computed tomography (CT) scan or magnetic resonance imaging (MRI).

### 2.5 Data extraction

To ensure the accuracy and reliability of the data, two independent reviewers (YS and JX) performed data extraction for each eligible study based on the pre-specified inclusion and exclusion criteria. Any discrepancies in the extracted data were resolved by consensus through discussion between the two reviewers or resolved by a third researcher (XM). The extracted information included study characteristics, baseline demographic, clinical characteristics of the subjects, interventions used, and safety-related outcomes.

### 2.6 Risk of bias assessment

To assess the quality of the included RCTs, we used the Cochrane Collaboration’s risk-of-bias tool (33), which provided an independent assessment of the risk of bias in the main outcomes of each trial. This tool assessed several key areas of bias, including random sequence generation (selection bias), allocation concealment (selection bias), blinding of participants and personnel (performance bias), blinding of outcome assessors (detection bias), incomplete outcome data (attrition bias), selective outcome reporting (reporting bias), and other potential sources of bias. Three levels - high, unclear and low risk - to assess the quality of the evidence. Two of the authors (QZ and JX) independently assessed the risk of bias and consulted a third reviewer (XM) in case of disagreement. By using a standardized and rigorous approach to assessing the risk of bias, we ensured that the included studies were of high quality and that the results of our meta-analysis were reliable.

### 2.7 Data synthesis and analysis

In our study, all statistical analyses were performed using the software package “Stata 17”. We presented estimates of the safety issues as a pooled proportion with a corresponding 95% confidence intervals (CIs). We used both the Higgins *I* ^2^ statistic and Cochran’s Q test to assess potential statistical heterogeneity among studies. An *I* ^2^ statistic greater than 50% was considered indicative of significant heterogeneity. The meta-analysis was conducted using a fixed-effects model. Sensitivity analyses were performed to assess the stability of the pooled effects. Subgroup analyses were performed according to different drug doses and control measures. *P* < 0.05 was considered statistically significant. We used a funnel plot to assess publication bias; if the funnel plot was symmetrical, there was no publication bias; otherwise, there was publication bias. Because of the subjectivity of the funnel plot, we also used Egger’s and Begg’s tests to test for publication bias. If the p-value of Egger’s or Begg’s test was less than 0.05, it indicated the presence of bias; otherwise, there was no bias.

## 3 Results

### 3.1 Search results

**Figure 1:**
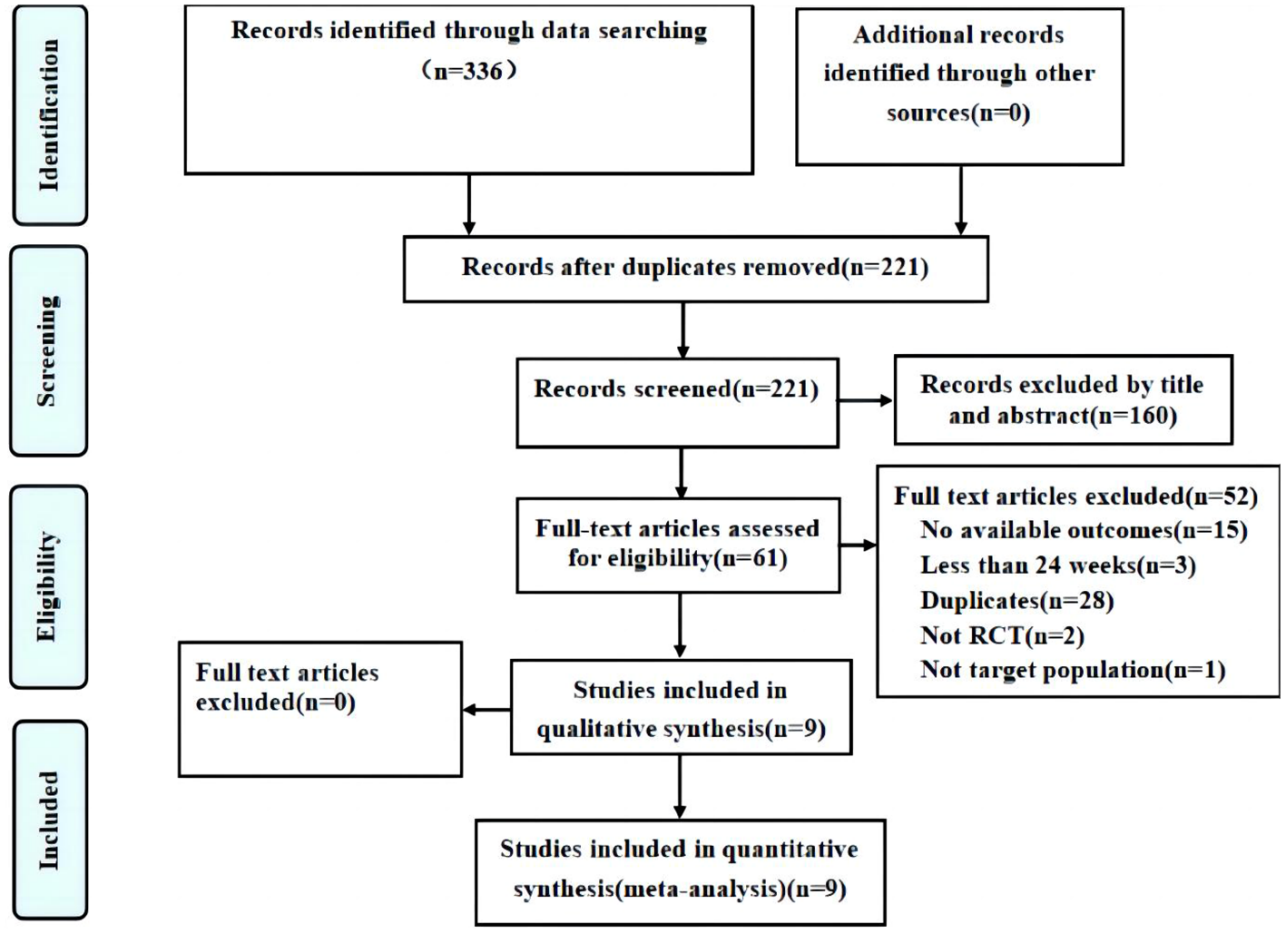
Flow diagram of the study.

The selection process is summarized in **Figure 1**. Our search strategy identified a total of 336 records, of which 115 were excluded due to duplication and 160 were excluded on the basis of titles and abstracts. We then assessed 61 full articles for eligibility and finally included nine studies that met our inclusion criteria. Of the 52 excluded records, 28 were duplicates, 15 had no results, 3 had a study duration of less than 24 weeks, 2 were non-RCTs and one did not include patients with T2D; the remaining nine studies (29–31, 34–39) satisfied the inclusion criteria and were included.

### 3.2 Study characteristics

In our study, the baseline characteristics of the study population were shown in **Supplementary Table S1**. The included trials ranged in duration from 26 to 72 weeks and compared tirzepatide with basal insulin (glargine or degludec), selective GLP1-RA (dulaglutide or semaglutide once weekly) or placebo. There were 6,828 patients with T2D in the tirzepatide group and 3,043 in the control group. Mean HbA1c at baseline ranged from 7.83% to 8.60% and the age of the study population ranged from 44.7 to 63.7 years. The duration of T2D or obesity ranged from 4.5 to 14.80 years in the included studies.

### 3.3 Risk of bias assessment

The Cochrane Collaboration’s Risk of Bias Tool including the risk-of-bias summary and risk-of-bias graph was used to assess the methodological quality, as shown in **Supplementary Figures S1**, **S2**. Random sequence generation, allocation concealment, blinding of participants and personnel, and blinding of outcome assessors were clearly presented in six studies (29–31, 34, 37, 39). Blinding of participants and personnel was not performed in three studies (35, 36, 38).The selection bias, detection bias, attrition bias, reporting bias and other biases were low.

### 3.4 Pancreatitis

**Figure 2:**
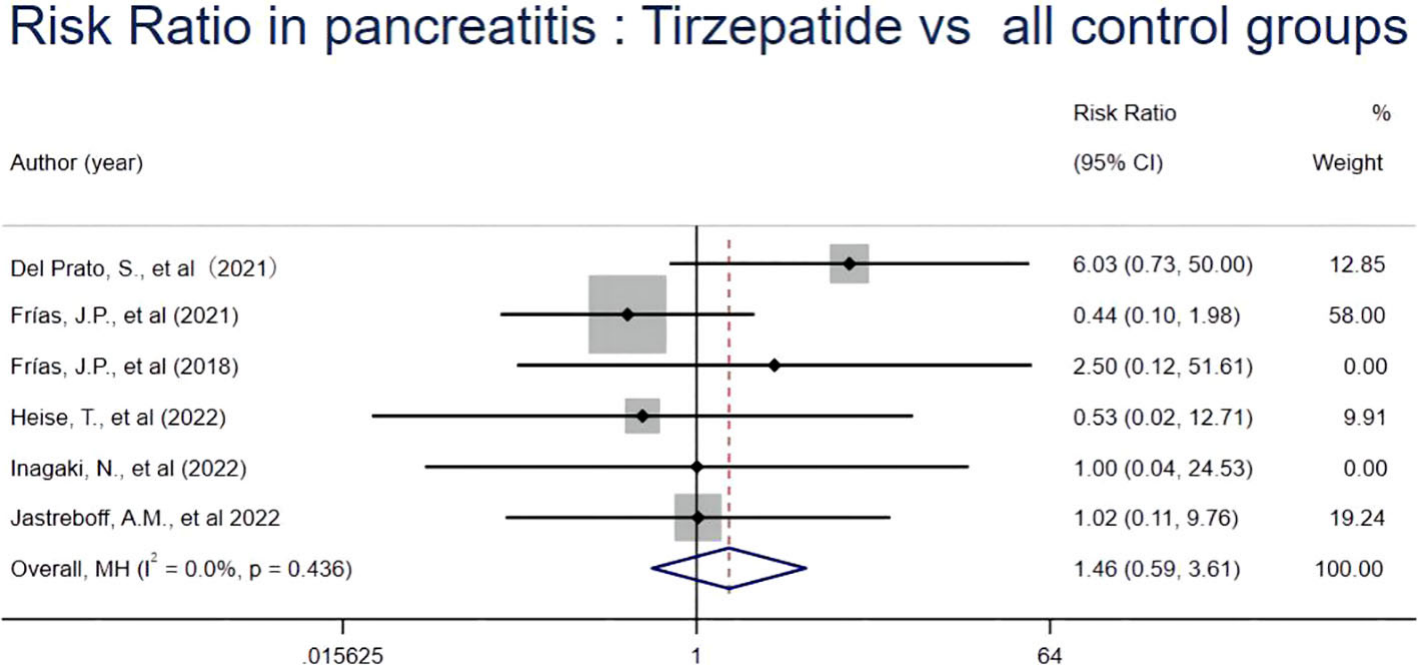
Risk Ratio in pancreatitis: tirzepatide vs control groups.

In our meta-analysis of nine RCTs, six trials reported pancreatitis events that occurred during the study period. Compared with all control groups (basal insulin (glargine or degludec), selective GLP1-RA (dulaglutide or semaglutide once weekly) and placebo), our results showed that tirzepatide was not significantly associated with an increased risk of pancreatitis (RR 1.46, [95% CI] 0.59 to 3.61; *I* ^2 = ^0.0%, *p* = 0.436), as shown in **Figure 2**. Furthermore, our subgroup analysis showed no significant difference in the risk of pancreatitis between different drug doses and control measures (placebo or basal insulin group and selective GLP1-RA group), as shown in **Supplementary Figures S3**-**S5**. These results suggest that tirzepatide was not associated with a significant risk of pancreatitis in patients with T2D and obesity.

### 3.5 Gallbladder or biliary disease

In our meta-analysis of nine RCTs, eight studies documented occurrences of gallbladder or biliary disease within the study duration. When compared with all control groups (including placebo, basal insulin, and GLP1-RA), our analysis did not show a statistically significant association between tirzepatide use and the incidence of cholelithiasis, cholecystitis, biliary diseases, or the the composite of gallbladder or biliary diseases, as shown in **Supplementary Figure S6**.

However, in our subgroup analysis, when compared to the placebo or basal insulin groups, we observed a statistically significant association between tirzepatide and an increased risk of the composite of gallbladder or biliary diseases (RR1.97, [95% CI] 1.14 to 3.42; *I* ^2 = ^0.0%, *p* = 0.558). No significant association was found for cholelithiasis or cholecystitis. These results were shown in **Supplementary Figures S7A, B**. Compared with GLP1-RA, our analysis did not find a significant association between tirzepatide and the risk of cholelithiasis, cholecystitis, biliary diseases and the composite of gallbladder or biliary diseases, as shown in **Supplementary Figure S8**.

Subgroup analysis by dosage showed that the use of 10 mg tirzepatide was associated with an increased risk of the composite of gallbladder or biliary disease (RR 1.91, [95% CI] 1.14 to 3.19; *I* ^2 = ^0.0%, *p* = 0.880) compared with all control groups. This association is shown in **Supplementary Figure S9A**. However, no significant association was observed between tirzepatide use and the incidence of the composite of gallbladder or biliary disease for the 5mg and 15mg dose groups compared with all control groups, as shown in **Supplementary Figures S9B, C**.

**Figure 3:**
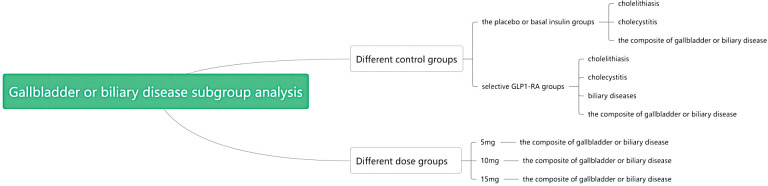
Flowchart of subgroup analysis.

To improve the readability of the article, I used flowcharts to illustrate my ideas for subgroup analysis in **Figure 3**.

### 3.6 Sensitivity analyses

We performed sensitivity analyses in our meta-analysis to assess the robustness of our findings by excluding individual studies one at a time for both pancreatitis and gallbladder or biliary disease. Our results showed no significant differences in the changes, indicating the stability of our findings.

### 3.7 Publication bias

Because the interpretation and judgement of funnel plots depends on the symmetry of the study results, funnel plots are not drawn in this paper when the number of studies is less than 10, as the credibility of the funnel plot is low and may not provide accurate information. Begg’s funnel (**Supplementary Figure S10**) and Begg’s and Egger’s tests were performed, the p-value was more than 0.05, which indicated that there was no publication bias.

## 4 Discussion

To our knowledge, this is the first study to systematically assess the safety issues of tirzepatide, specifically regarding pancreatitis and gallbladder or biliary disease, in patients with T2D and obesity. Our findings suggest that tirzepatide is safe for pancreatitis, but it may increase the risk of the composite of gallbladder or biliary diseases in RCTs. These results provide valuable insights into the safety profile of tirzepatide and may inform clinical decision-making regarding its use in patients with T2D and obesity.

Only one study evaluated the safety of tirzepatide (pancreatitis and gallbladder or biliary disease) (31). A systematic review and meta-analysis included patients with T2D and found no significant association between tirzepatide and an increased risk of pancreatitis or cholelithiasis. Our conclusions were consistent for pancreatitis, but we did a more systematic analysis for gallbladder or biliary disease. Firstly, the study included not only T2D, but also obese patients. Second, other gallbladder or biliary diseases (cholecystitis and biliary diseases) were added and an outcome analysis of the composite of gallbladder or biliary diseases was performed. Finally, taking into account the latest available research and the mechanism of action of GIP and dual agonists, subgroup analyses were performed according to different control groups and clinically meaningful results were obtained.

The limited statistical power and small sample size when analysing independent diseases may have contributed to the inability to detect a significant association between tirzepatide use and these individual diseases. However, when these independent diseases were combined into the composite of gallbladder or biliary diseases, the larger sample size allowed for increased statistical power. In addition, combining multiple diseases may reveal stronger associations, possibly due to shared pathological mechanisms or disease processes. Thus, analysing multiple diseases together may reveal a stronger association.

The composition of tirzepatide includes both GIP and GLP-1 RA, which have been implicated in gallbladder or biliary disease. GLP-1 has been shown to impair gallbladder motility and contractility by inhibiting the secretion of cholecystokinin, a hormone involved in the digestion and absorption of fat, which may contribute to the development of gallbladder or biliary disease (40). In addition, GIP has recently been reported to play a role in gallbladder relaxation (41). These findings suggest that the components of tirzepatide may have a direct effect on gallbladder or biliary disease, which may explain the increased risk observed in our study.

This study has several important strengths. Firstly, this is the first systematic review and meta-analysis to consolidate the results of RCTs to thoroughly evaluate the association between tirzepatide and safety issues, specifically pancreatitis and gallbladder or biliary disease. Secondly, our study included a larger and more diverse population, comprising individuals with both T2D and obesity. This approach adds to the generalizability of our findings. Thirdly, our results revealed the potential impact of the duration of tirzepatide treatment on the development of gallbladder or biliary disease, providing valuable insights into the time-dependent effects and considerations for the safe use of this drug.

There are some limitations to meta-analysis: (1) Bias and confounding: Each study has its own unique biases and confounding factors. (2) Variability in data quality: Different studies may have variations in data quality. (3) Publication bias: Published trials tend to report positive results resulting in overly optimistic or inaccurate effect estimates. (4) Heterogeneity: Heterogeneity between studies may affect the interpretation and generalisation of results.

We must therefore acknowledge some limitations. Firstly, the studies included in our analysis were not specifically designed to evaluate the safety profile of tirzepatide, particularly with regard to pancreatitis and gallbladder or biliary disease, which may have led to incomplete ascertainment of the relevant outcomes. Secondly, some gallbladder or biliary diseases were not a pre-specified safety outcome in the included studies, leading to potential under-reporting of these outcomes. However, we believe that any under-reporting is unlikely to have biased the associations found in our study. Thirdly, the basic characteristics of the populations included in our study were somewhat heterogeneous, which may have introduced some bias when analyzed together. Therefore, caution should be exercised when interpreting the results, and further research is needed to explore potential confounding factors and elucidate the true nature of the associations between tirzepatide and safety outcomes.

## 5 Conclusion

Tirzepatide might be safe for pancreatitis but increase the risk of gallbladder or biliary diseases in patients with T2D and obesity. Our findings suggest that physicians should be concerned that safety issue in patients treated with tirzepatide in clinical practice.

## Data availability statement

The original contributions presented in the study are included in the article/**Supplementary Material**. Further inquiries can be directed to the corresponding authors.

## Author contributions

Conception and design of the study: SL and HF. Screening of databases, extraction and analysis of the study data: QZ, JX, and XM. Drafting of the initial manuscript: QZ and JX. Verification of the study methodology: YS, XM. Revision of the manuscript: SL, JX. All authors contributed to the manuscript and approved the submitted version.
