# Corrigendum: Safety issues of tirzepatide (pancreatitis and gallbladder or biliary disease) in type 2 diabetes and obesity: a systematic review and meta-analysis

**DOI:** 10.3389/fendo.2024.1342543

**Published:** 2024-03-14

**Authors:** Qingyue Zeng, Jiao Xu, Xingyu Mu, Yi Shi, Hong Fan, Shuangqing Li

**Affiliations:** ^1^ General Practice Ward/International Medical Center Ward, General Practice Medical Center, West China Hospital, Sichuan University, Chengdu, Sichuan, China; ^2^ Department of Pulmonary and Critical Care Medicine, West China Hospital, Sichuan University, Chengdu, China

**Keywords:** dual agonists, incretin based therapy, tirzepatide, type 2 diabetes, obesity, glucagon-like peptide-1 receptor agonists

In the published article, there was an error in affiliation 1. Instead of “Department of General Practice Medicine Center, West China Hospital, Sichuan University,Chengdu, China,”, it should be “General Practice Ward/International Medical Center Ward, General Practice Medical Center, West China Hospital, Sichuan University, Chengdu, Sichuan, China.”

In the published article, there was an error in the Funding statement. The statement was originally as follows: “The authors declare financial support was received for the research, authorship, and/or publication of this article. This study was supported by the National Clinical Research Center for Geriatrics, West China Hospital, Sichuan University (Grant Number Z2021JC005).” The correct Funding statement appears below.

“The authors declare financial support was received for the research, authorship, and/or publication of this article. This study was supported by the Sichuan Science and Technology Program (No. 2017SZYZF0002 and No. 2021YFH0168).”

The authors apologize for these errors and state that this does not change the scientific conclusions of the article in any way. The original article has been updated.

