# Supplementary material for: Safety issues of tirzepatide (pancreatitis and gallbladder or biliary disease) in type 2 diabetes and obesity: a systematic review and meta-analysis
Source: Front Endocrinol (Lausanne). 2023 Oct 16;14:1214334. doi: 10.3389/fendo.2023.1214334 (PMC10613702; doi:10.3389/fendo.2023.1214334)
Supplement: Supplementary file 1 [file DataSheet_1.docx]

**The Supplementary Material**


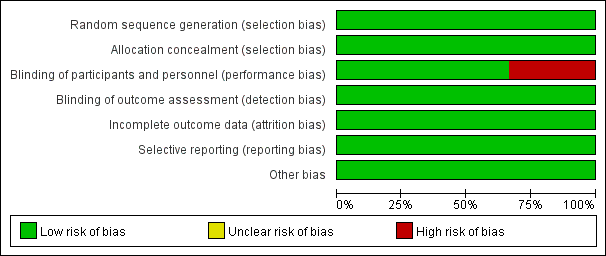


Figures S1. Risk of bias graph


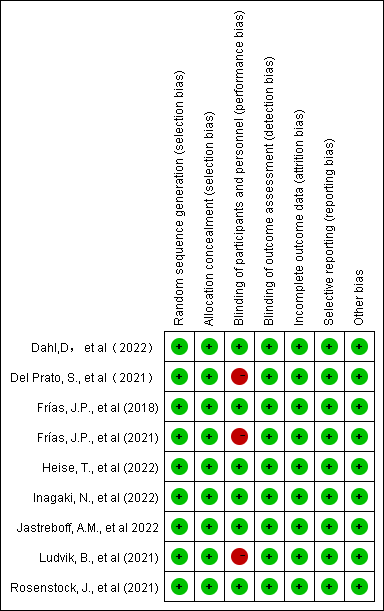


Figures S2. Risk of bias summary


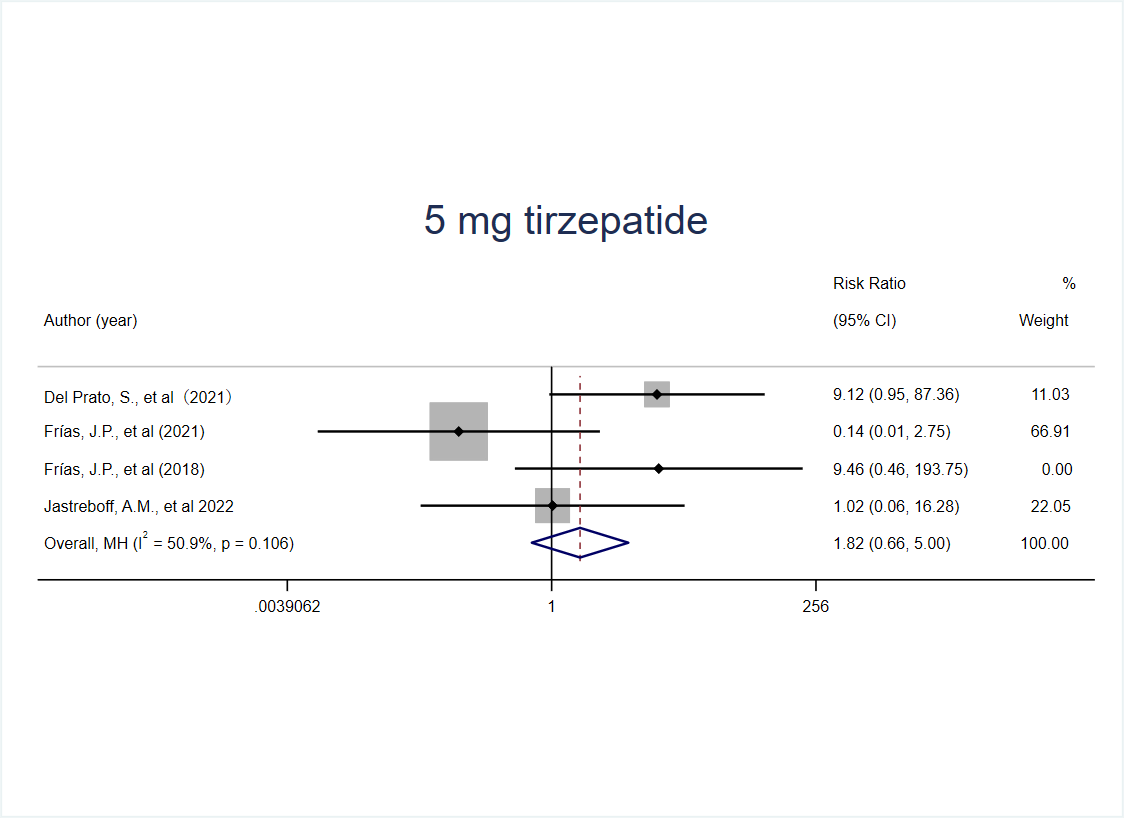


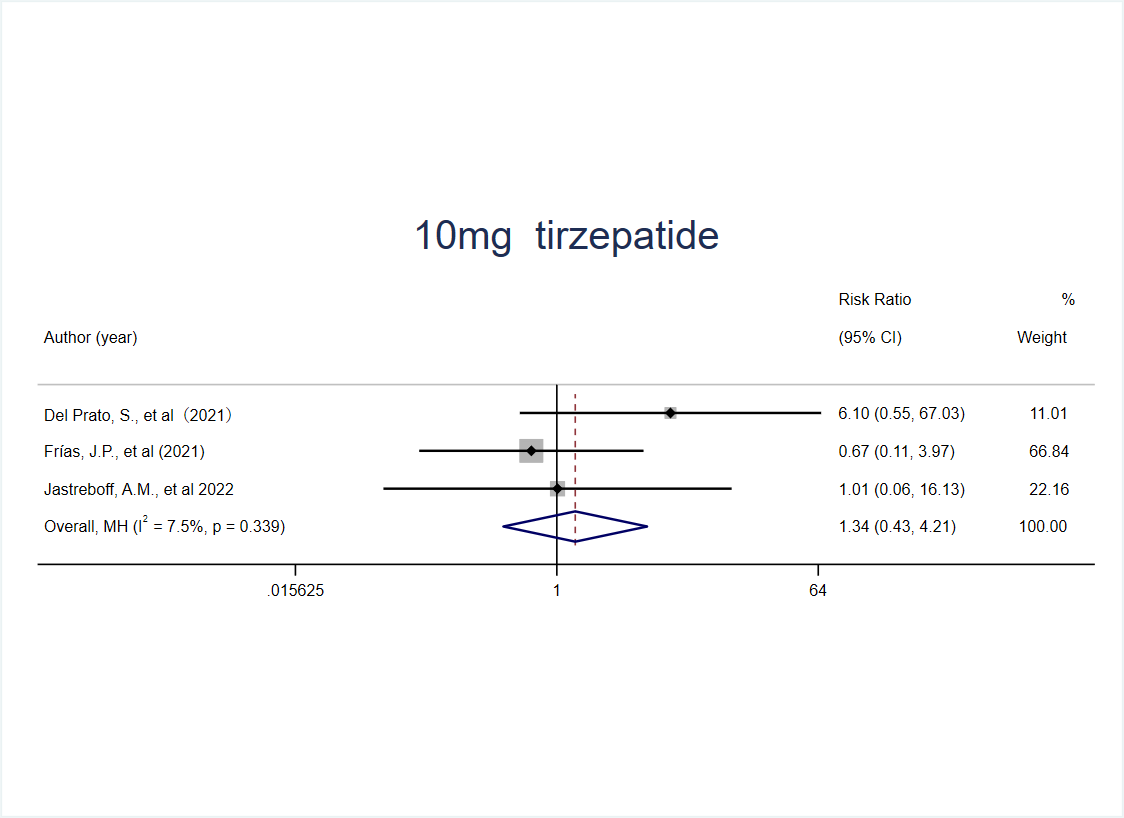


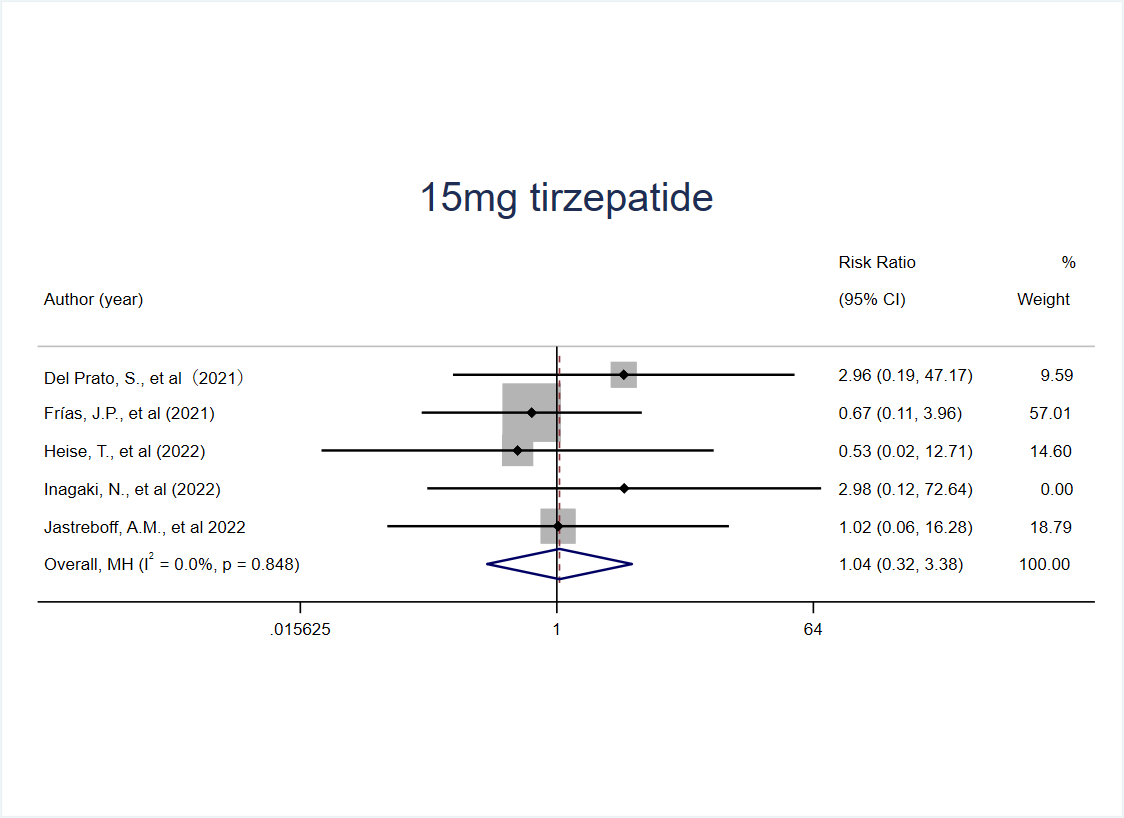


Figures S3. Risk Ratio in pancreatitis: different doses tirzepatide vs all control groups


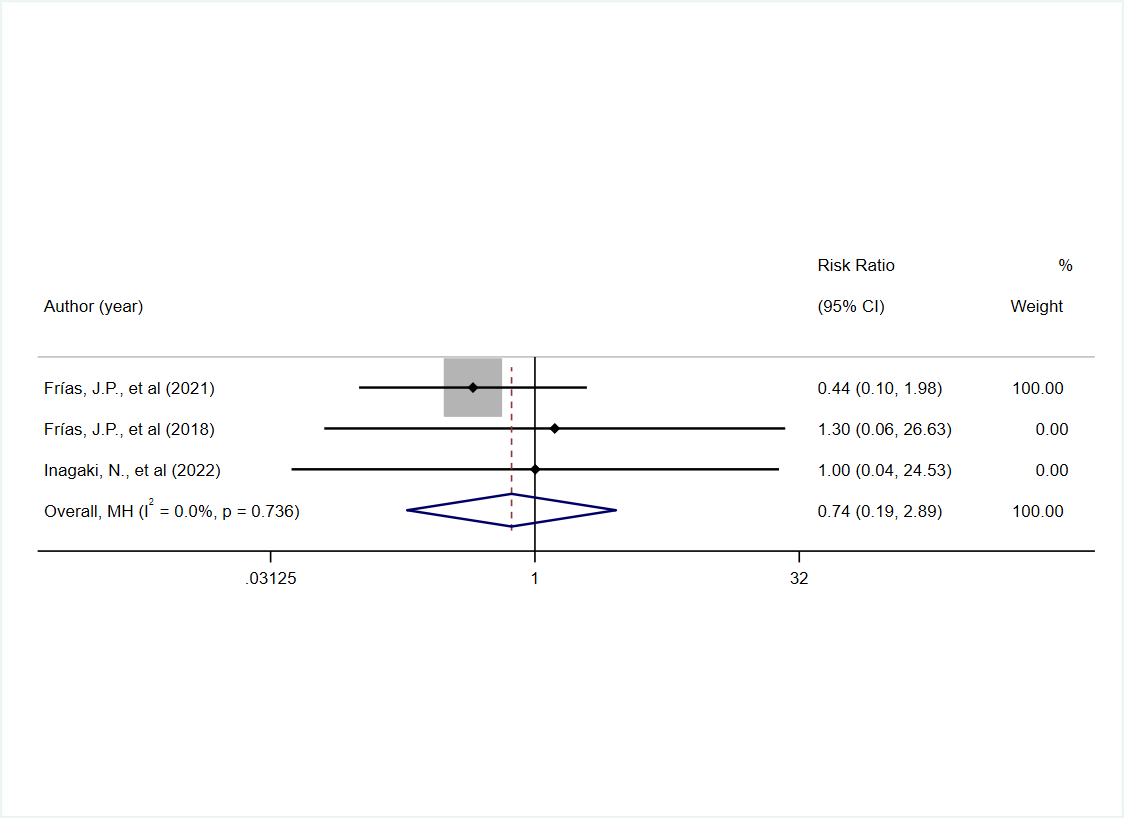


Figures S4. Risk Ratio in pancreatitis: tirzepatide vs selective GLP1-RA


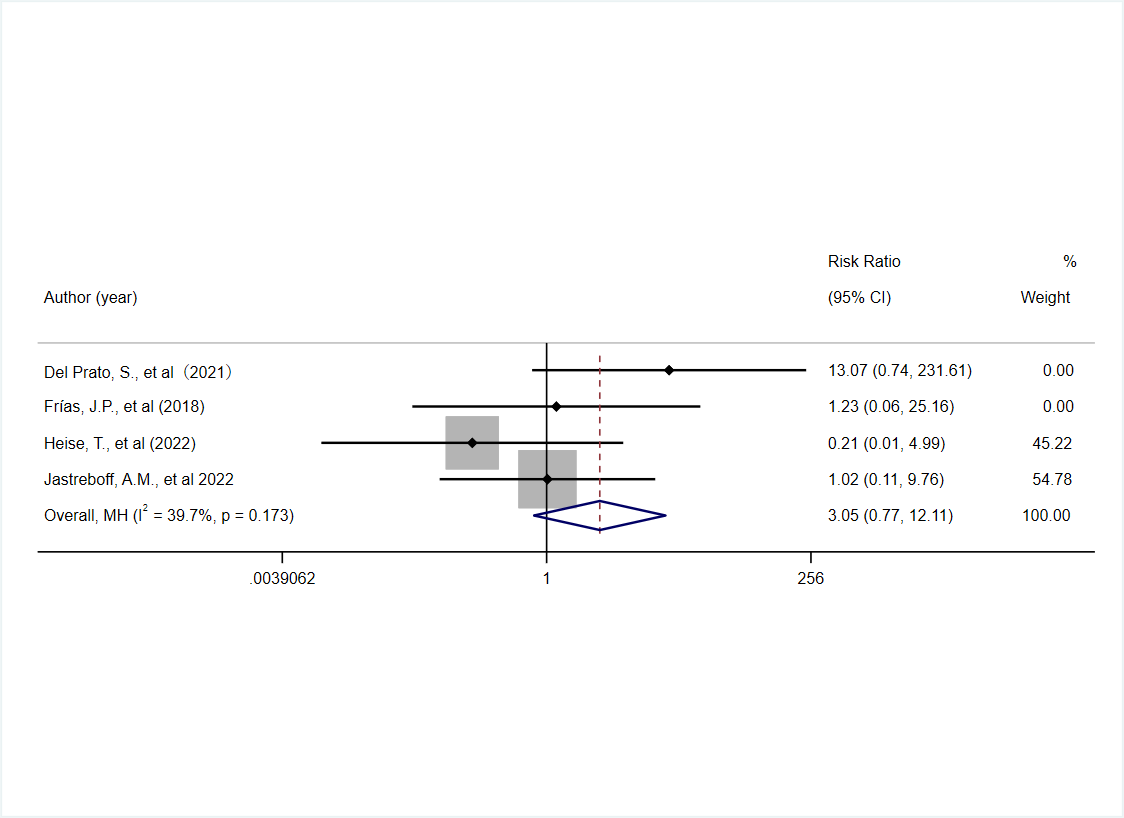


Figures S5. Risk Ratio in pancreatitis: tirzepatide vs placebo or basal insulin


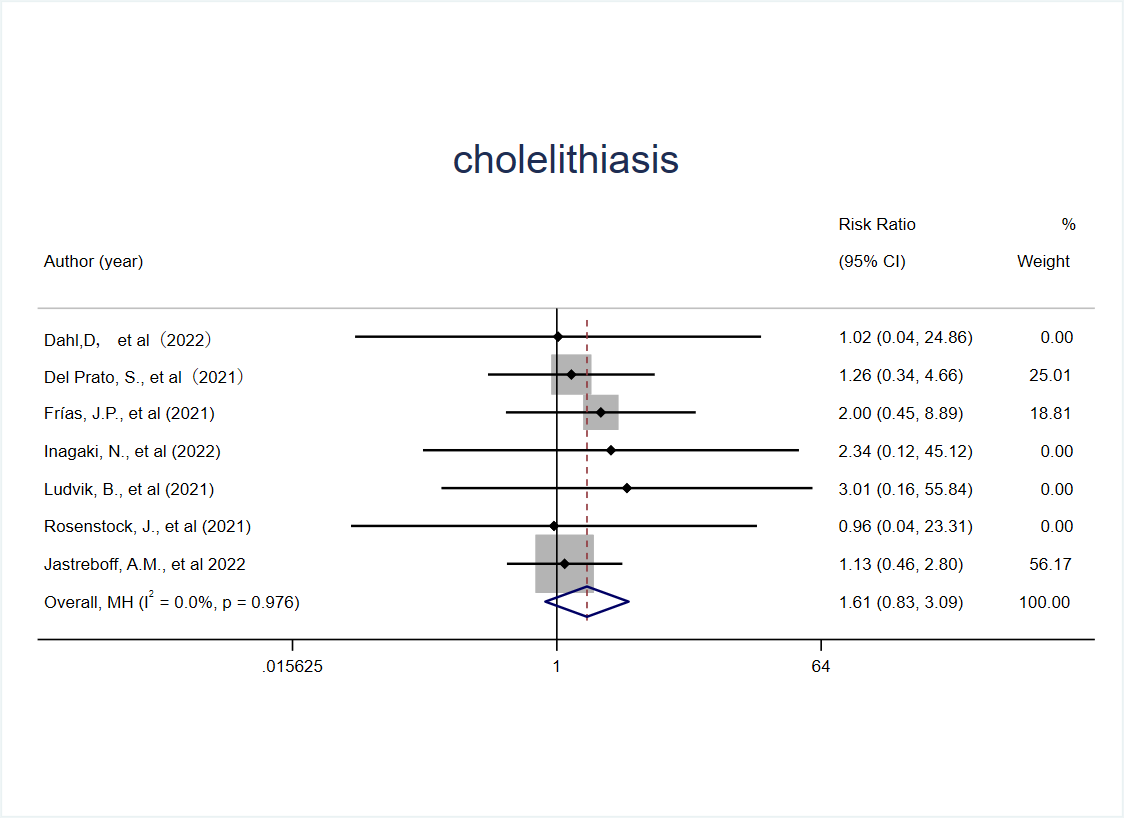


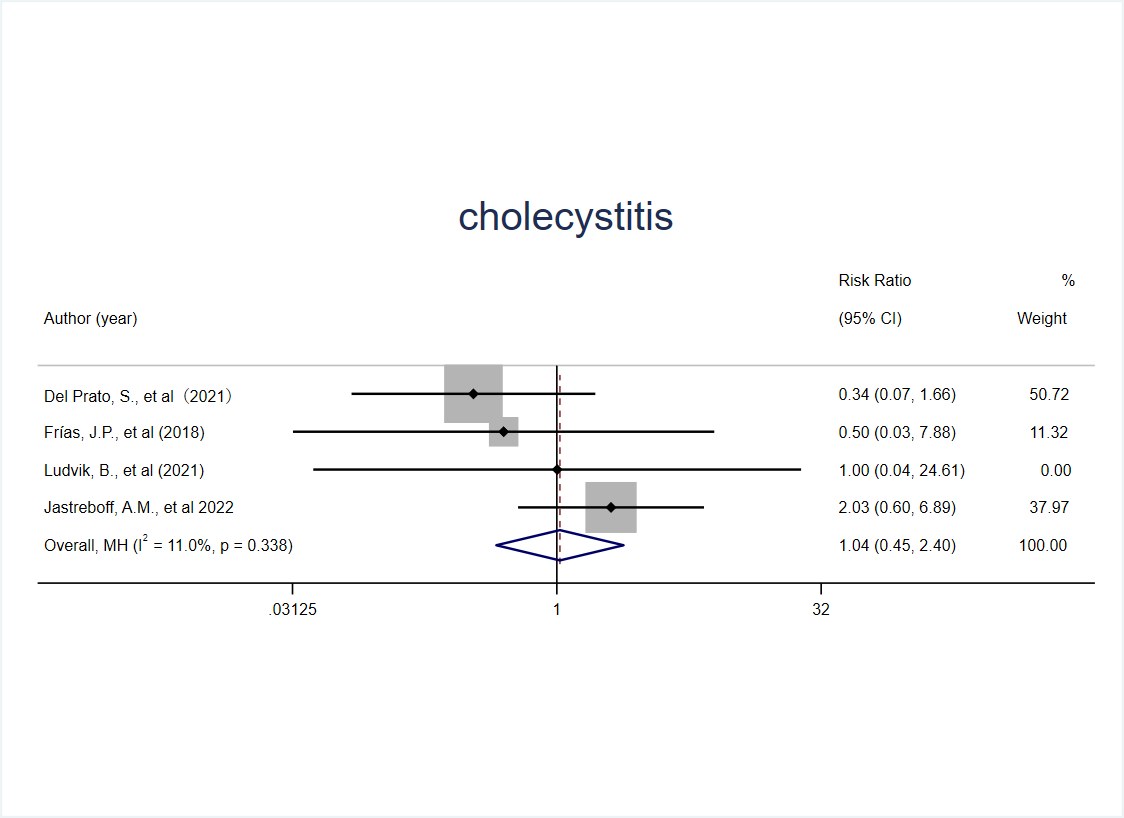


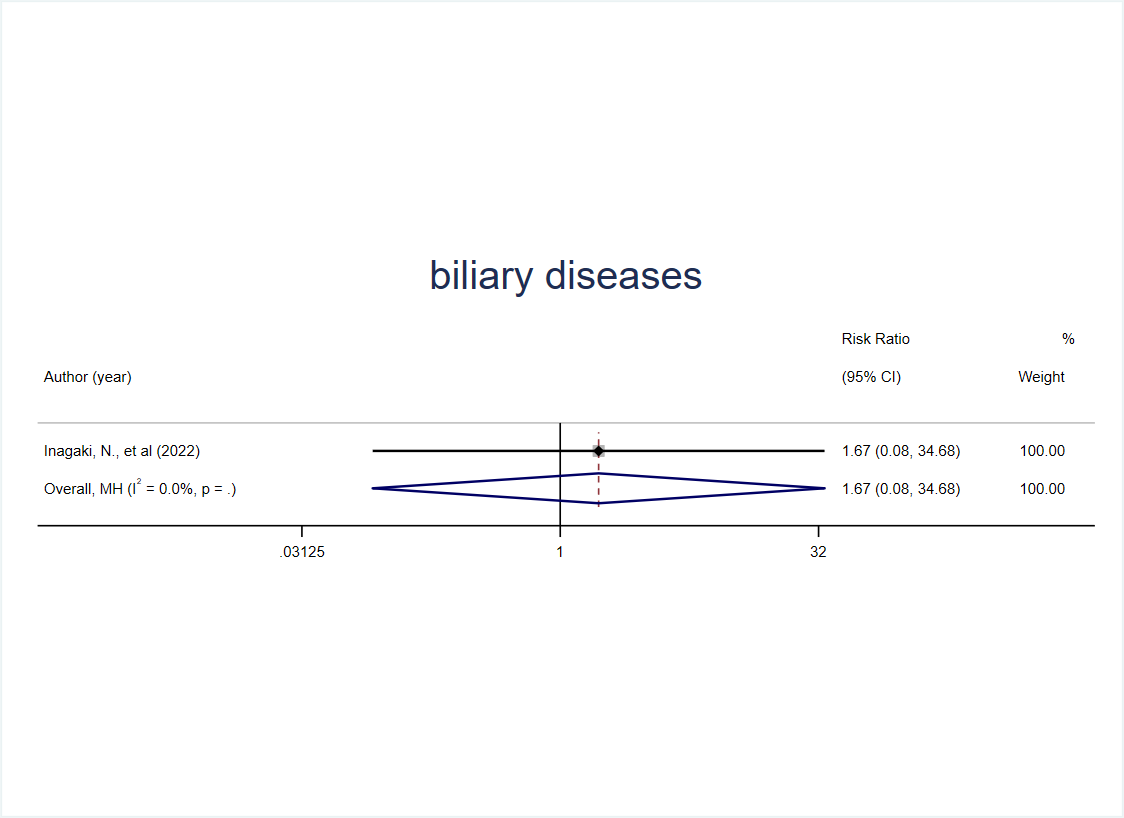

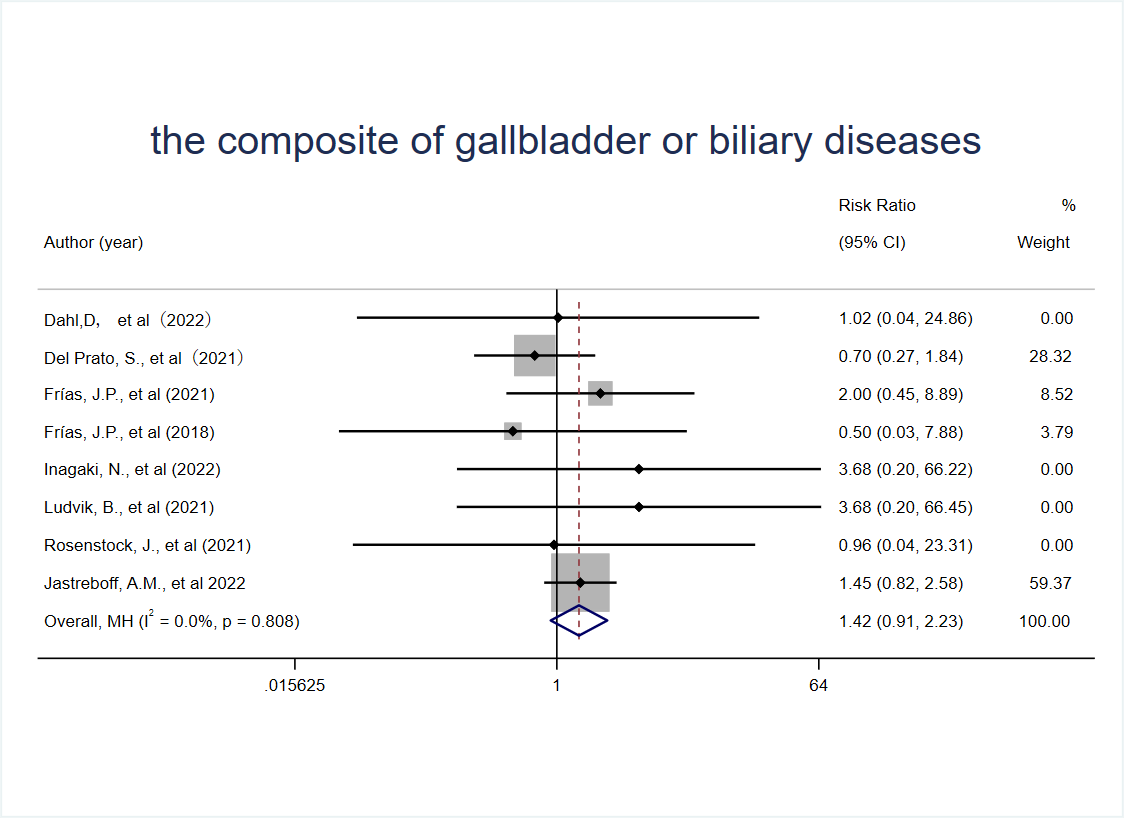


Figure S6 : Risk Ratio of gallbladder or biliary diseases: tirzepatide vs all control groups


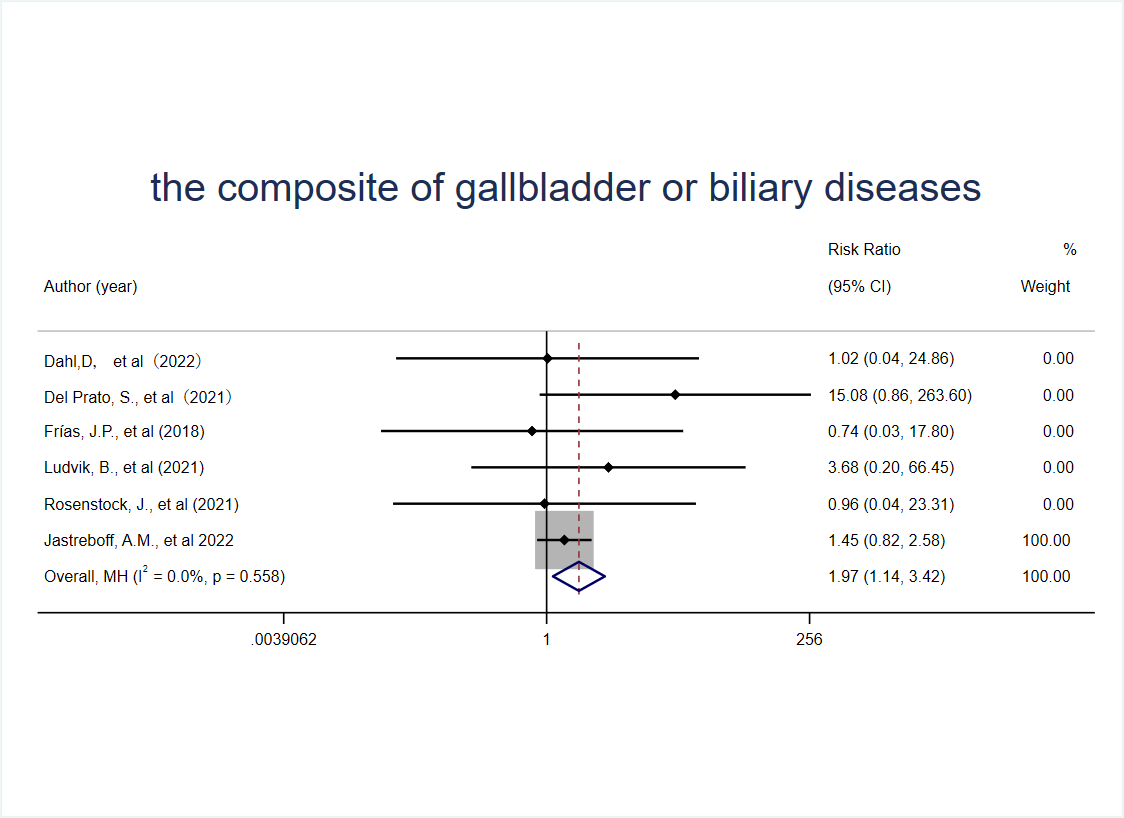


Figures S7A. Risk Ratio of the composite of gallbladder or biliary diseases: tirzepatide vs placebo or basal insulin


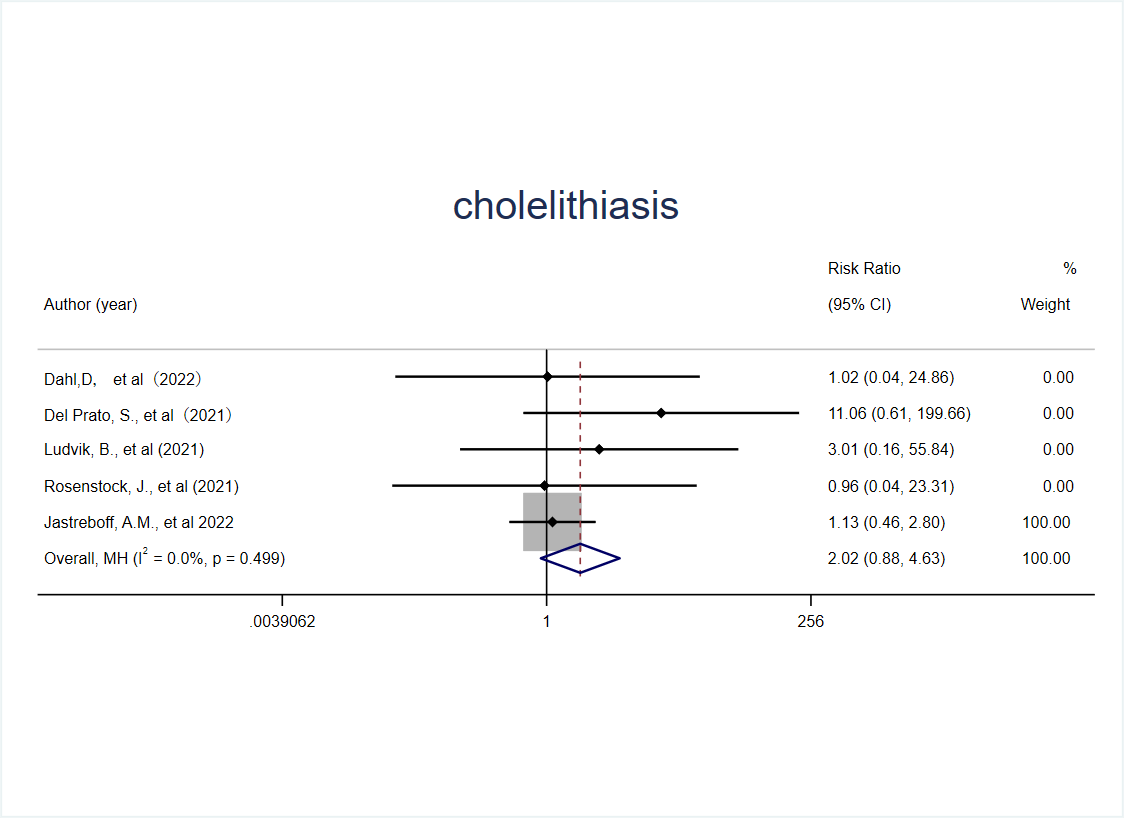

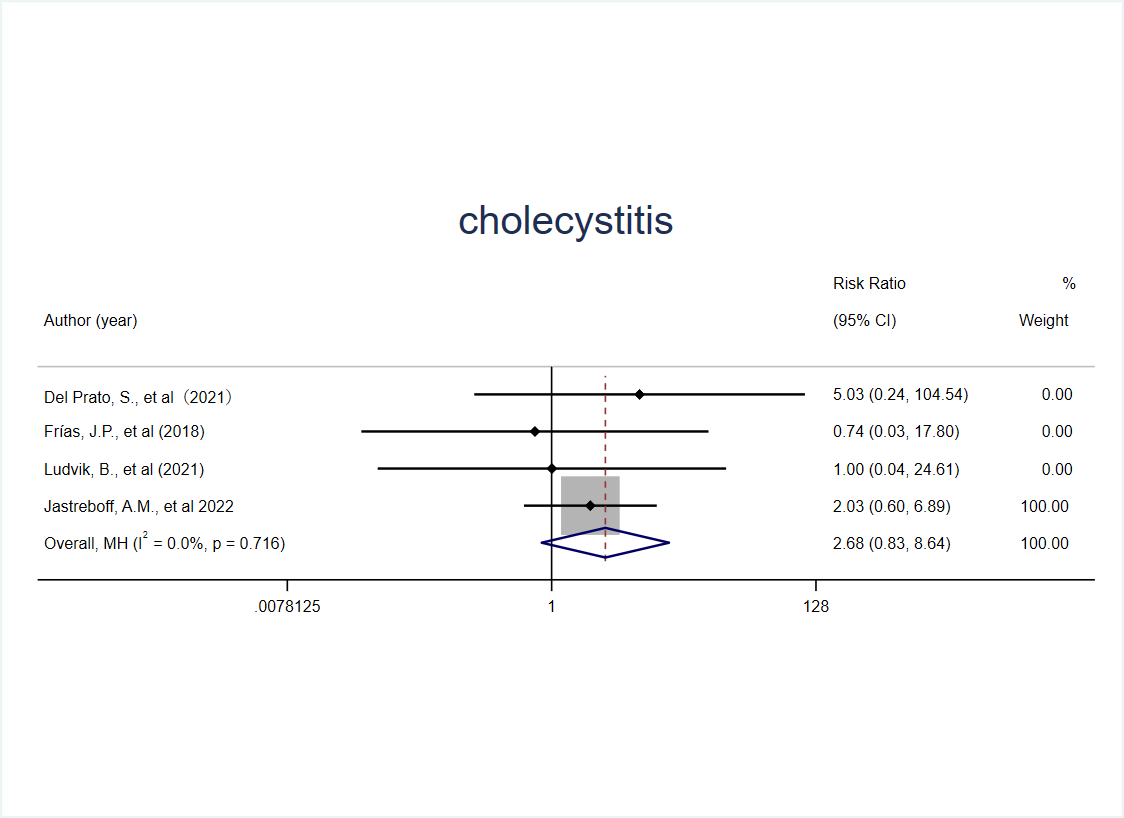


Figures S7B. Risk Ratio of cholelithiasis and cholecystitis: tirzepatide vs placebo or basal insulin


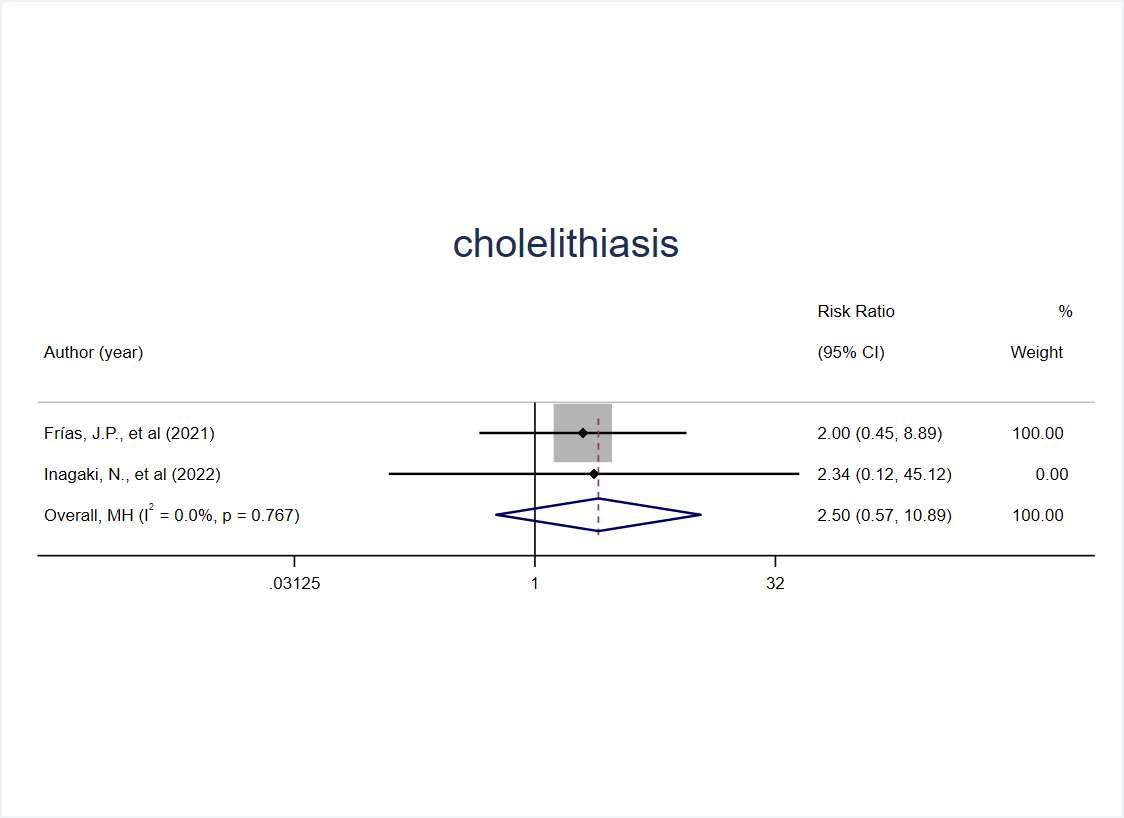


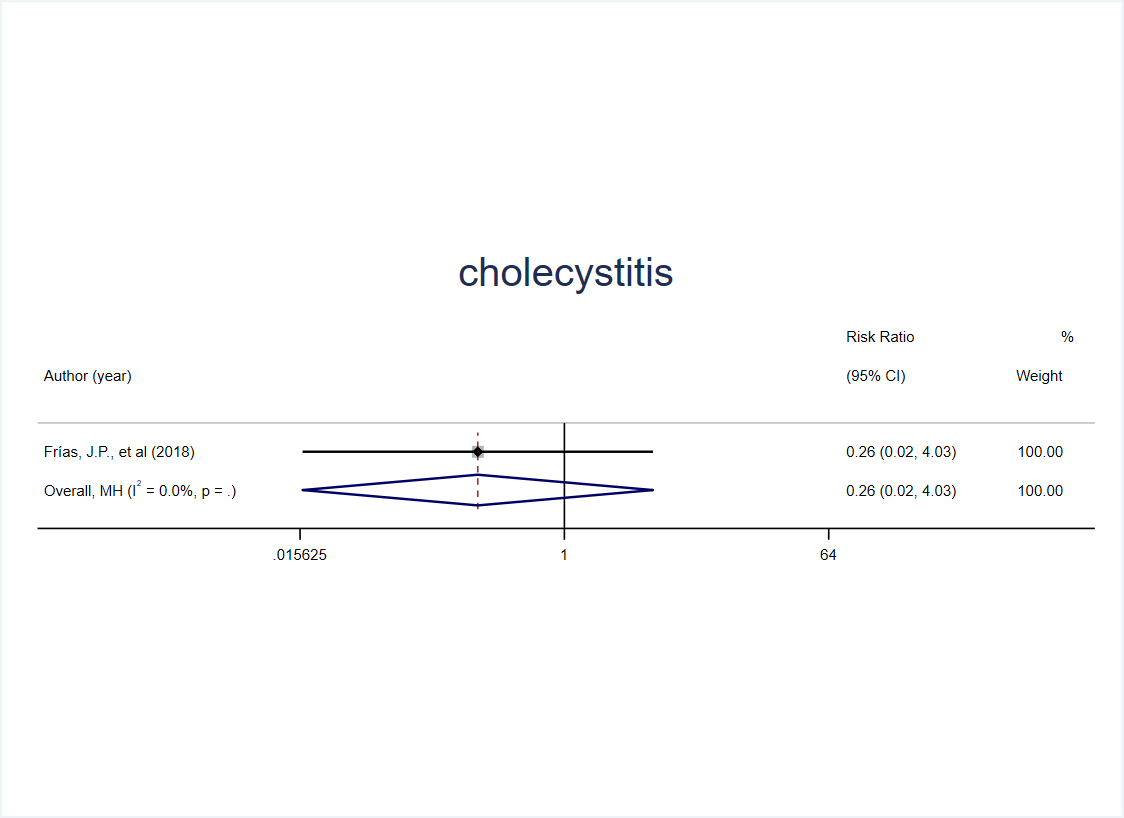


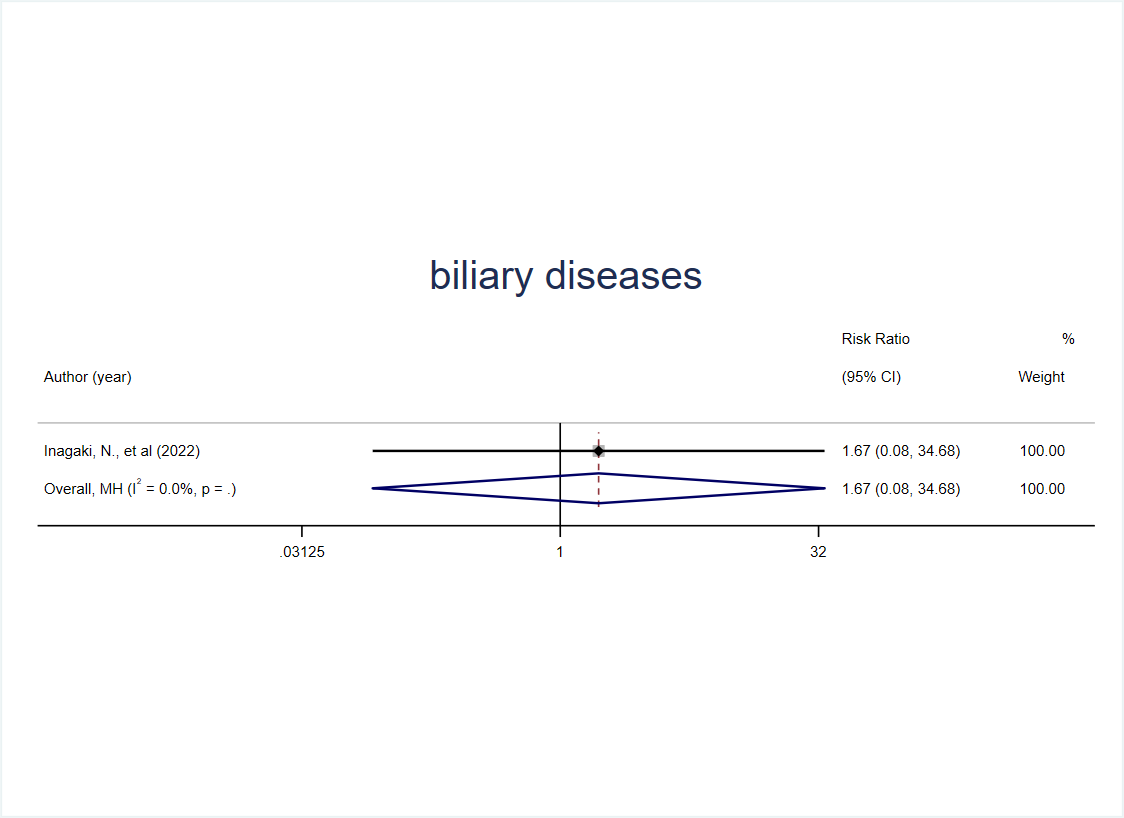


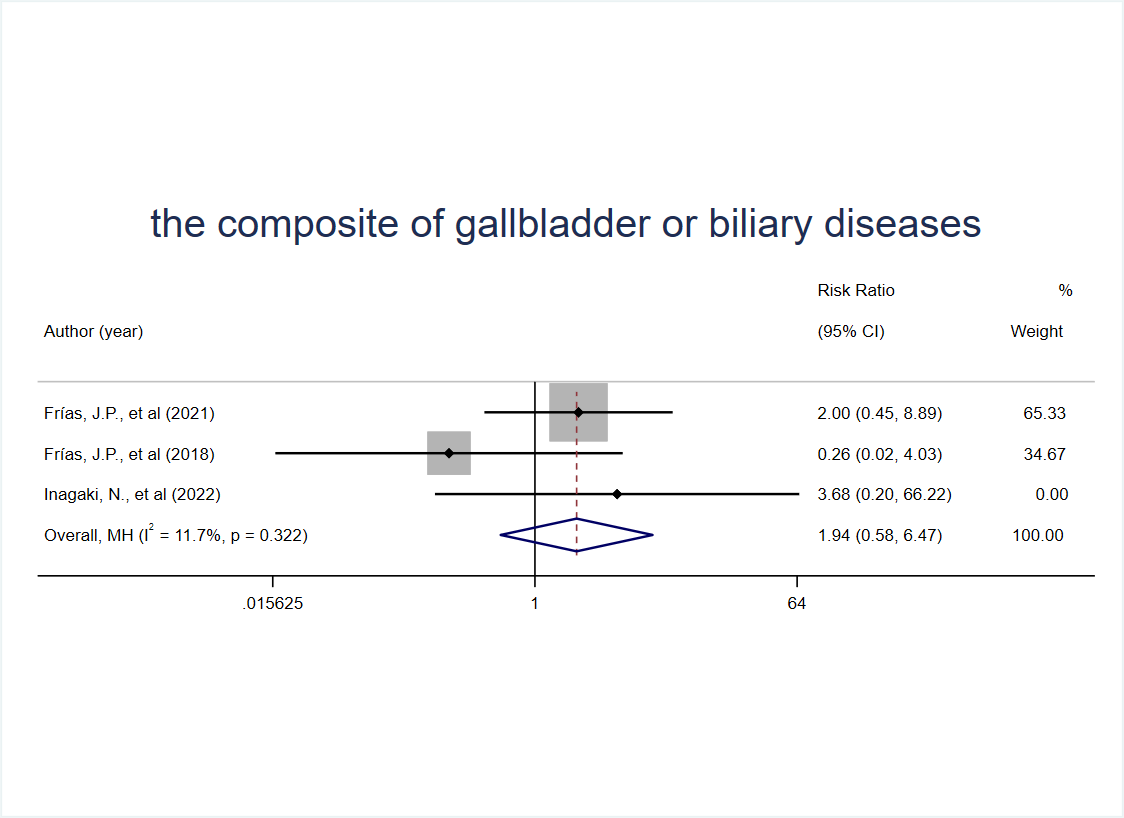


Figures S8: Risk Ratio of gallbladder or biliary diseases: tirzepatide vs selective GLP1-RA


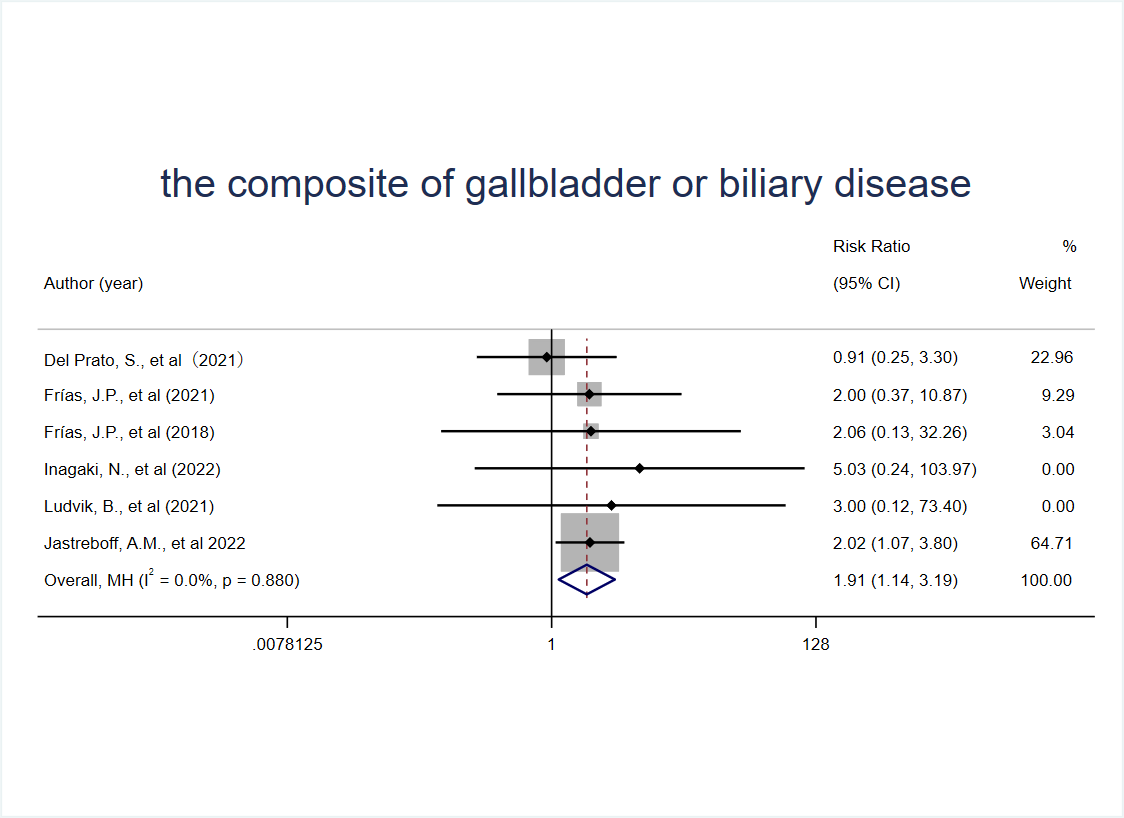


Figures S9A. Risk Ratio of the composite of gallbladder or biliary diseases: 10mg tirzepatide vs all control groups


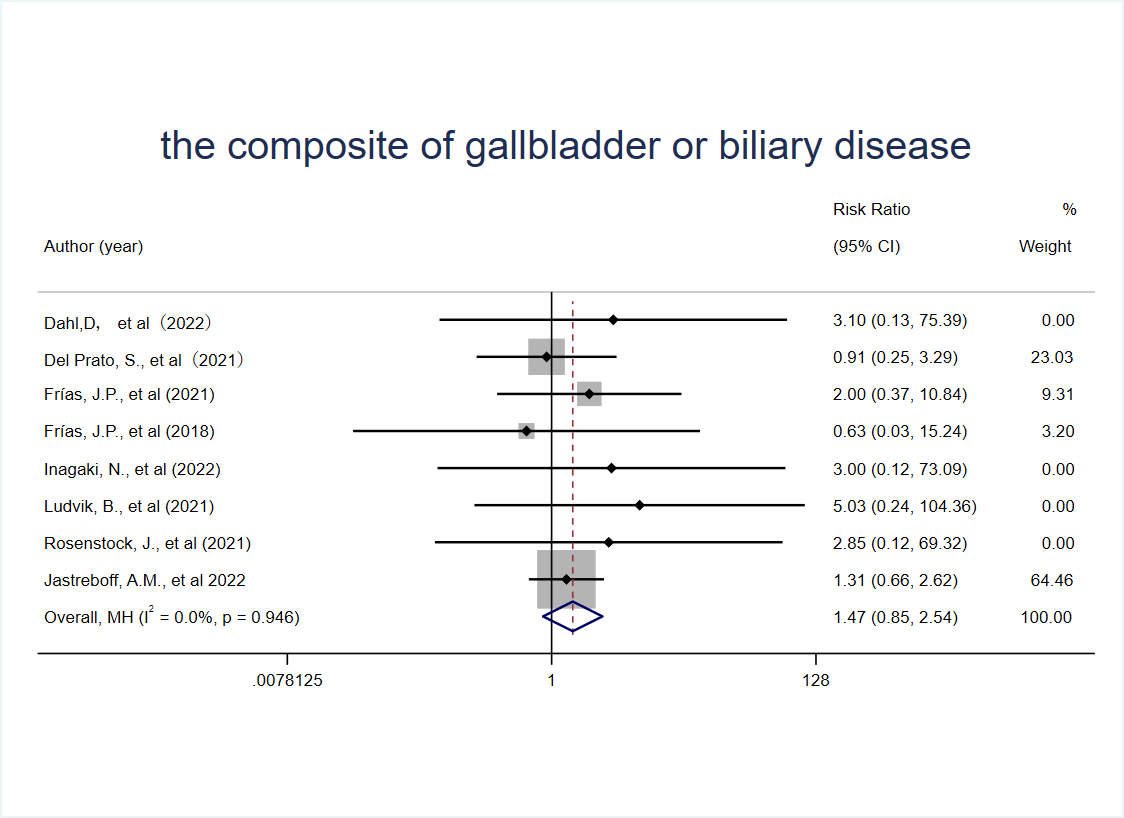


Figures S9B. Risk Ratio of the composite of gallbladder or biliary diseases: 5mg tirzepatide vs all control groups


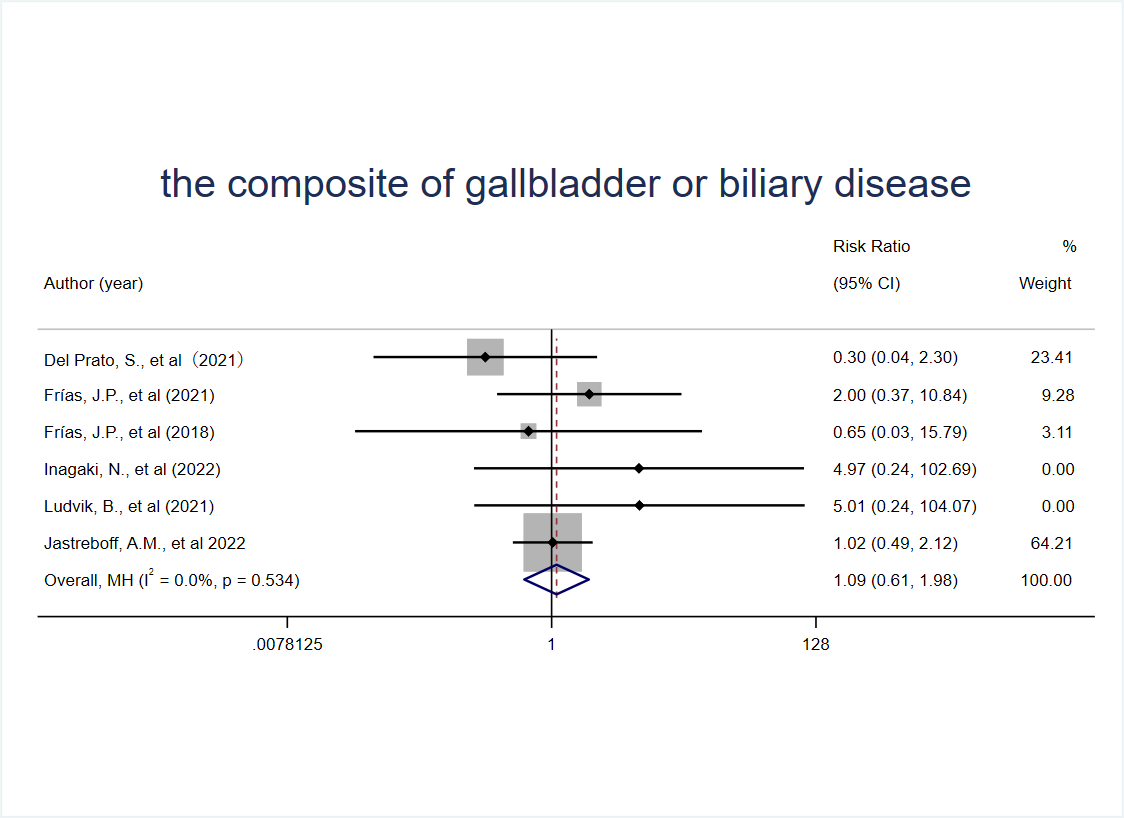


Figures S9C. Risk Ratio of the composite of gallbladder or biliary diseases: 15mg tirzepatide vs all control groups


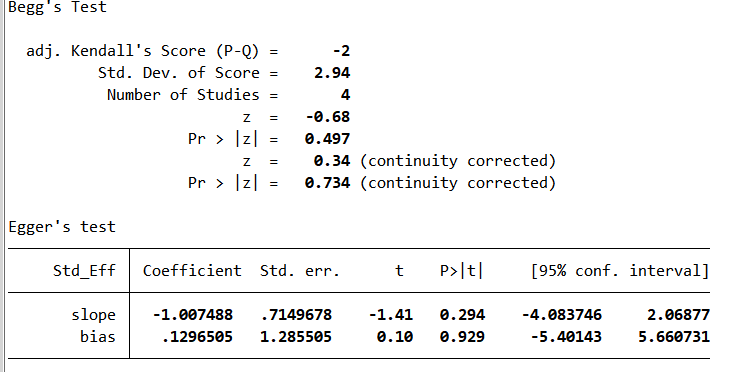


Figures S10. Begg’s Test and Egger’s Test
